# Performance Evaluation and Mechanism Study of Seawater-Based Circulatory Fracturing Fluid Based on pH-Regulated WormLike Micelles

**DOI:** 10.3389/fchem.2022.848269

**Published:** 2022-04-26

**Authors:** Haifeng Tang, Jiamei Song, Min Zhao, Zhiyang Zhang, Weixing Liu, Zhihu Yan

**Affiliations:** ^1^ Jiangsu Key Laboratory of Marine Bioresources and Environment/Jiangsu Key Laboratory of Marine Biotechnology, Jiangsu Ocean University, Lianyungang, China; ^2^ Co-Innovation Center of Jiangsu Marine Bio-industry Technology, Jiangsu Ocean University, Lianyungang, China; ^3^ School of Ocean Engineering, Jiangsu Ocean University, Lianyungang, China

**Keywords:** pH-sensitive surfactant, salt-resistant, wormlike micelles, fracturing fluid, recycling

## Abstract

In this article, a novel salt-resistant pH-sensitive surfactant *N*-carboxystearamido methanesulfonic acid (MSA) was designed and synthesized. The rheological properties of the MSA/CTAB mixed system prepared using seawater were evaluated, and the variation laws of the related rheological parameters were discussed. The relevant fracturing technical parameters of the MSA/CTAB mixed system were comprehensively evaluated. The wormlike micelles formed by the non-covalent binding of MSA and CTAB molecules can resist the electrostatic effect of inorganic salts in the seawater. Meanwhile, the MSA/CTAB mixed system has an excellent pH response and revealed that the change from wormlike micelles to spherical micelles leads to the decrease of the apparent viscosity and the transition from Maxwell fluid to Newton-type fluid. Furthermore, the MSA/CTAB mixed system has excellent cyclic fracturing performance, which can meet the dual requirements of fracturing fluid cost and performance of offshore oilfield, and has a good application prospect.

## Introduction

Hydraulic fracturing technology is the preferred stimulation method for developing offshore low-permeability reservoirs (Guo et al., 2022; Memon et al., 2022). Conventional freshwater-based fracturing fluids are limited by space, transportation, and cost and cannot meet the needs of large-scale offshore fracturing (Cui et al., 2022). Therefore, it is of great theoretical and practical significance to study the seawater-based cyclic fracturing fluid system for low-cost, green, and efficient development of offshore low-permeability reservoirs (Zhao et al., 2019; Othman et al., 2021). The clean fracturing fluid has the advantages of thickening agent recycling, reservoir protection, and convenient operation, and is suitable for constructing seawater-based recycling fracturing fluid system (Shibaev et al., 2021).

Since the first appearance of the clean fracturing fluid in 1997, many scholars have carried out in-depth research on filtration, sand-carrying, temperature resistance, and formation of the clean fracturing fluid, which has significantly improved the properties of the clean fracturing fluid (Tian et al., 2021; Yang et al., 2022). However, the gel-breaking effect can be improved by adding a gel-breaking agent. The first type is redox agents or particular bacteria to break the molecular structure of the surfactant, resulting in a thickening agent that cannot be recycled (Mao et al., 2022). The second type is to change the micelle shape with the help of alcohol, alkanes, and surfactant. The thickener can be recycled, but it has some disadvantages such as uncontrollable gel-breaking time, damaging reservoir, difficult separation, and high cost (Tang et al., 2021; Xue et al., 2021). Therefore, it is a critical problem to realize reversible gel breaking with controllable time, complete gel breaking, and low cost for constructing a clean fracturing fluid system. To solve this problem, this article aims to realize the reversible gel breaking of clean fracturing fluid by introducing intelligent control switch, compared with intelligent control switch such as light, oxidation, CO_2_, and temperature; pH switches are time-controlled, thoroughly regulated and cost-effective, with good biocompatibility and high surface activity, so pH-sensitive surfactant is suitable as thickeners for clean fracturing fluids (Liu et al., 2021a; Yu et al., 2021; Mu et al., 2022).

Many researchers have studied pH-sensitive surfactants extensively, but most of the results have been obtained at room temperature and in pure water (Fu et al., 2021; Jiao et al., 2021). The seawater-based clean fracturing fluid is tough to construct, mainly because of the high salinity of seawater, which is 30,000–40,000 mg/L and contains a lot of Ca^2+^ and Mg^2+^ plasma (Sun et al., 2019; Zhang et al., 2019). On the one hand, high salinity induces the transition from high viscoelastic wormlike micelles to low viscoelastic layered structures through electrostatic action. On the other hand, it reduces the repulsive force between wormlike micelles. The two reasons both degrade the performance of the clean fracturing fluid.

**SCHEME 1:**
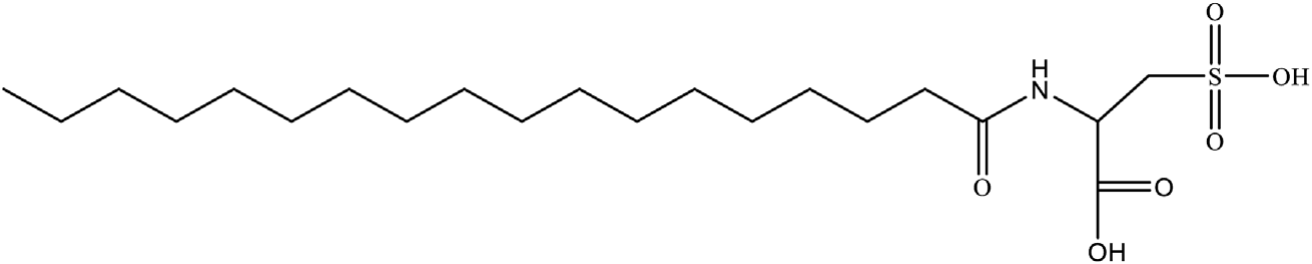
Molecular structure of MSA.

In this article, a novel salt-resistant pH-sensitive surfactant *N*-carboxystearamido methanesulfonic acid (MSA) was designed and synthesized. The molecular structure of MSA is shown in Scheme 1. Then the rheological properties of the mixed system prepared using seawater were evaluated and the variation laws of the related rheological parameters were discussed. After that, because of the presence of carboxylic acid as a pH-regulating functional group on MSA molecular head, the rheological properties of the mixed solution were further tested. Finally, the relevant fracturing technical parameters of the mixed system, such as high temperature and shear resistance, gel breaking, and sand carrying capacity, were comprehensively evaluated.

## Experimental

### Synthesis and Structural Characterization of MSA

DL-cystetic acid was purchased from Hubei Jusheng Technology Co., Ltd., China. Stearic acid was purchased from Sinopharm Chemical Reagent Co., Ltd., China. The synthesis steps of MSA have been described in detail in our published paper (Yan et al., 2020a). The 1H NMR spectrum was recorded in DMSO at 400 MHz on a Bruker AVANCE III HD NMR spectrometer (Bruker, Karlsruhe, Germany). The mass spectrum of MSA was acquired on an Agilent 6510 QTOF mass spectrometer (Agilent, Santa Clara, CA).

### Sample Preparation

All samples were prepared with Bohai seawater. The mole ratio of MSA/CTAB was fixed at 1:1. The mixed solution with a total concentration of 200 mmol L^−1^ was used as mother liquor, which was diluted with Bohai seawater to obtain the MSA/CTAB mixed solution with different concentrations. The pH value of the sample was adjusted by adding concentrated HCl solution and NaOH solid. All samples of viscoelastic fluid were placed in a constant temperature water bath at 25°C for 24 h before being tested.

### Rheology

The steady-state and dynamic rheological properties were measured with the Physica MCR 302 rotational rheometer made by Anton Paar Company of Austria. The upper plate was a standard Searle-type concentric cylinder with a radius of 40 mm and a cone angle of 1.5. Before the test, the parameters of the rheometer are calibrated, and then the cone plate is cleaned and zeroed. When testing, make sure that the solution fills the gap between the upper and lower plates. The shear rate range of the steady-state rheological test is 0.01–100 s^−1^. Before the dynamic rheological test, the dynamic stress-sweep test is needed to determine the linear viscoelastic region. The temperature of the sample should be maintained at about 25°C during the testing process.

### Cryo-TEM

The solution sample of 4 μL was transferred by the pipette gun and dropped on the 3.02-mm copper mesh with 200 meshes, and the excess sample was absorbed by the blotting paper. Then the copper mesh was put into liquid nitrogen to freeze quickly, and then the sample was cut off by vacuum spraying apparatus. Secondly, platinum was sprayed on the microstructure of the exposed sample, and then carbon was sprayed so that the micromorphology of the aggregate was printed on the platinum carbon film. Finally, the organic components of the replica films were corroded off, and the micromorphology of the samples was observed and photographed by a transmission electron microscope.

### Dynamic Light Scattering

The 10-ml quartz sample bottle was first treated with an acetone washing machine for 30 min, and then the dust particles were removed from the MSA/CTAB mixed solution by 0.22 μm syringe filter. Malvern Zetasizer Nano ZS90 laser light scattering instrument (wavelength 633 nm, scattering angle 90°, refractive index 1.332, temperature 20°C) was used in this experiment.

### Evaluation of Fracturing Fluid

**SCHEME 2:**
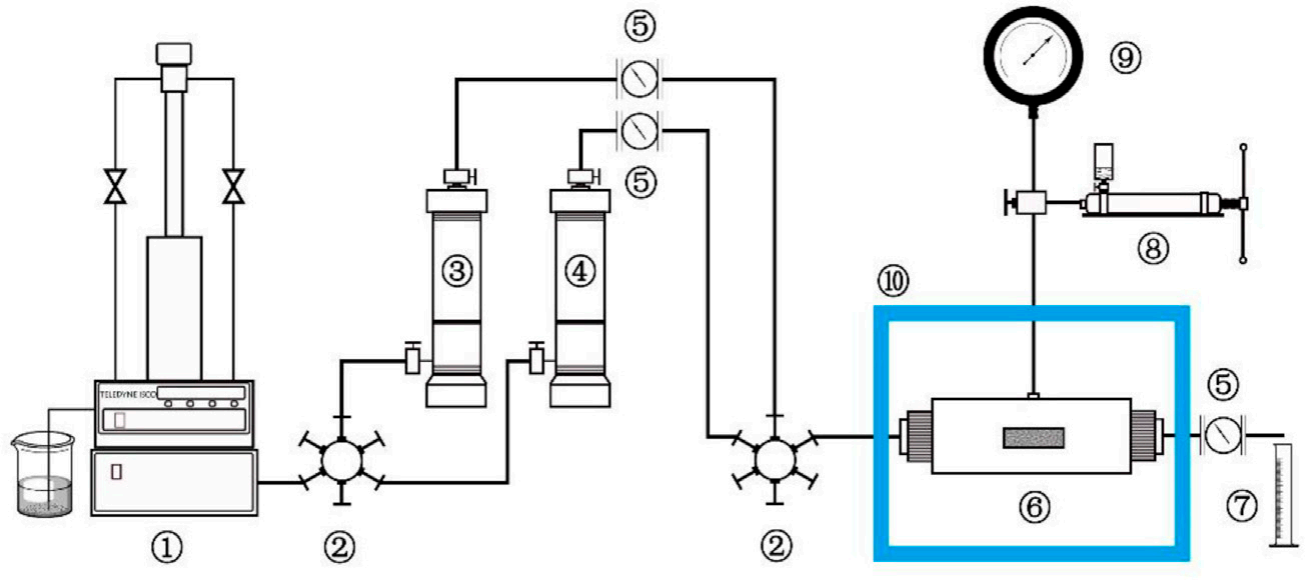
Schematic diagram of the experimental apparatus: 1-Pump, 2-Valve, 3,4-Intermediate container, 5-Flow meter, 6-Core container, 7-Measuring cylinder, 8-Hand pump, 9-Pressure gauge, 10-Thermostatic box.

The experimental methods of the evaluation indexes of fracturing fluid in this article were tested strictly according to China’s “General specification for fracturing fluids-SY/T6376—2008”, so they are not repeated here. In this article, the laboratory equipment for evaluating the cyclic utilization performance of fracturing fluid was designed and built. Therefore, the fracturing property of the MSA/CTAB mixed solution after several times of pH adjustments can be investigated whether it still meets the requirement of the fracturing standard. The schematic diagram of the experimental apparatus is shown in Scheme 2.

## Results and Discussion

### Structural Characterization

**FIGURE 1:**
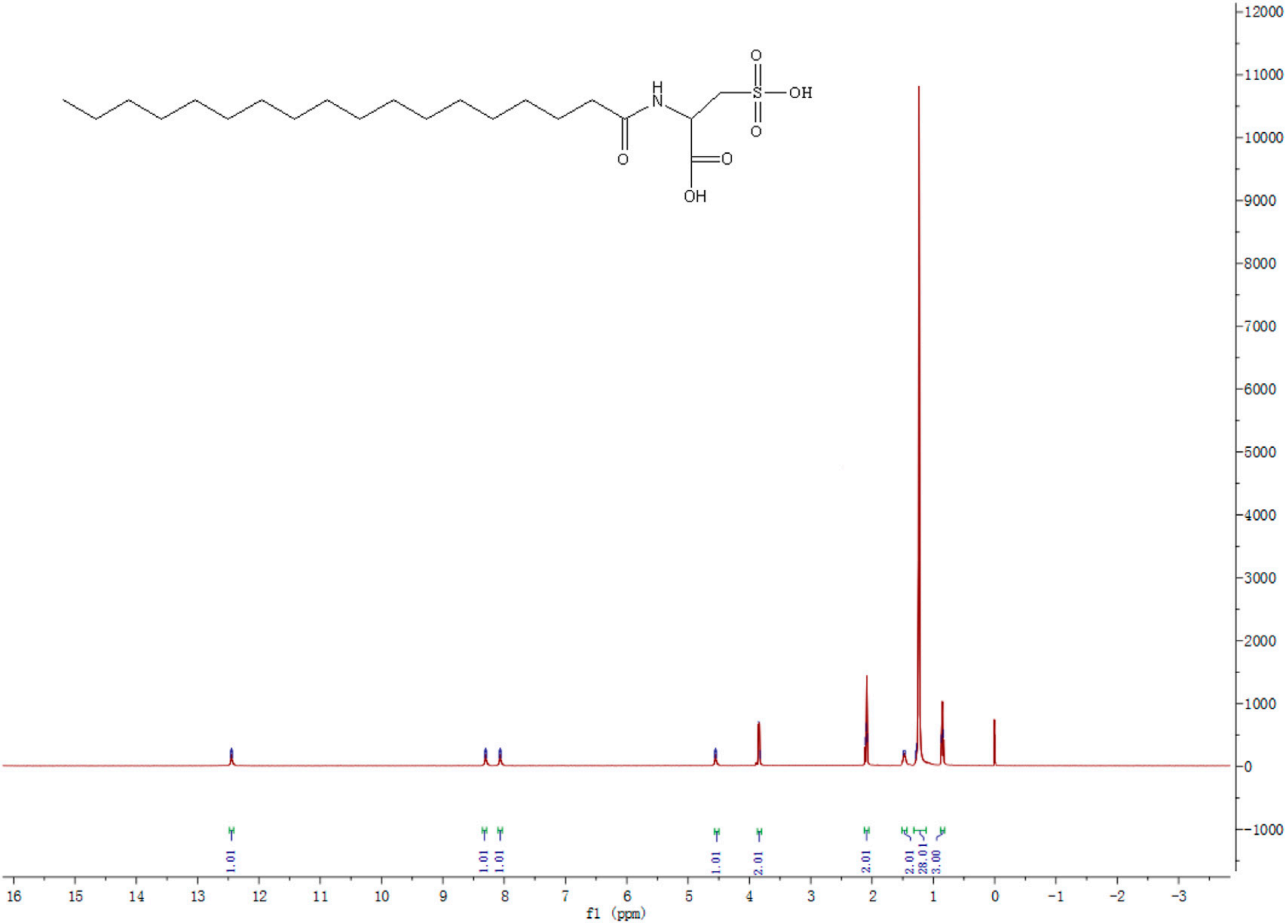
1H-NMR spectrum of MSA.

**FIGURE 2:**
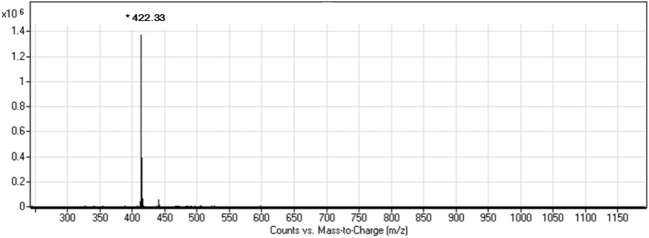
Mass spectrum of MSA.

Figure 1 depicts the 1H NMR (DMSO, 400 MHz, ppm) spectrum of MSA: *δ* = 0.87(3H, m, CH_3_), 1.25(28H, m, 14×CH_2_), 1.52(2H, m, CH_2_), 2.33(2H, q, CH_2_), 3.81(2H, s, CH_2_), 4.51(1H, s, CH), 8.12(1H, s, NH), 8.33(1H, s, OH), and 12.44(1H, s, OH). The mass spectrum of MSA (Figure 2) shows a molecular ion peak m/z = 422.3. According to the results of mass spectrum and 1H NMR, the target product MSA was prepared successfully according to the synthesis route of 2.1, and the purity of MSA was up to the test requirement.

### Rheological Behavior of the Viscoelastic Fluids

**FIGURE 3:**
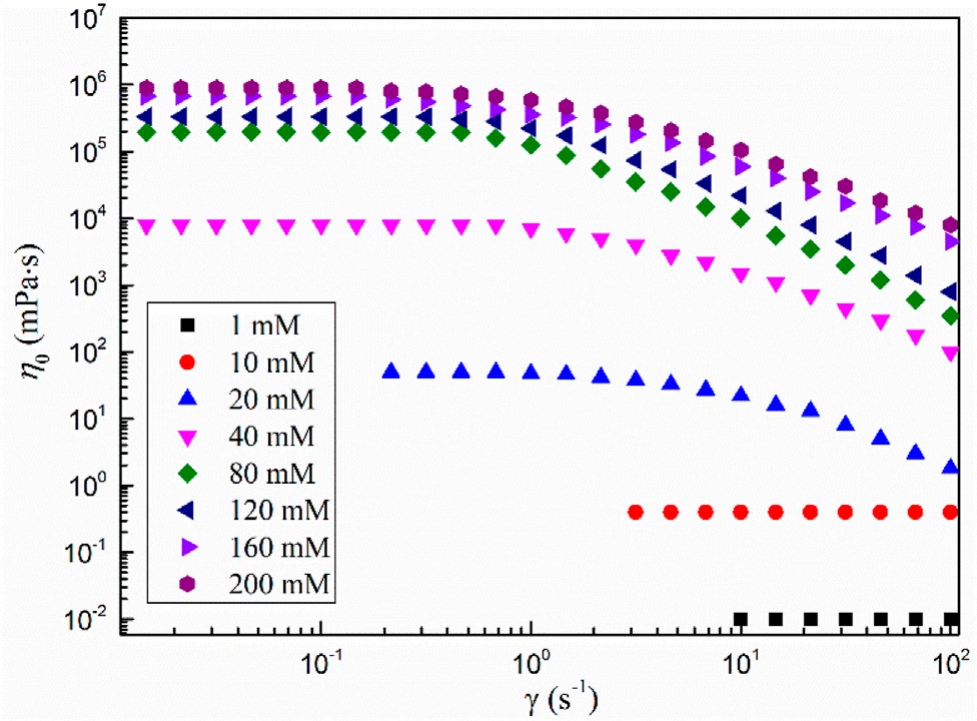
Shear viscosity versus shear rate curves of the MSA/CTAB system.

Figure 3 shows the shear viscosity versus shear rate curves of the MSA/CTAB system with different total concentrations. It can be seen from the diagram that the shear viscosity of the system is independent of the shear rate when the total concentration is less than 10 mmol·L^−1^, showing a significant Newton fluid character (Mushi et al., 2021). When extrapolating the viscosity curve to zero shear rate yields a zero shear viscosity *η*_0_ of 0.4 mPa·s, very close to the viscosity of water, which generally indicates that the solution is dominated by small spherical or short stick aggregates (Wang et al., 2021). When the total concentration was higher than 20 mmol·L^−1^, the solution showed different characteristic viscosity curves: Newton plateau appeared at the low shear rate, and shear thinning appeared after the shear rate reached the critical value. This behavior is considered an important sign of the formation of wormlike micelles, which indicates that the MSA/CTAB mixed system is a viscoelastic fluid with wormlike micelles as its internal structure (Danov et al., 2018). This is because at low shear rates the fluid is a three-dimensional network of wormlike micelles with a higher viscosity and a smaller change in shear rate. The rearrangement of wormlike micelles occurs when the higher shear force is enforced, the more the network structure of wormlike micelles is rearranged and the viscosity of the system is decreased (Feng et al., 2021).

**FIGURE 4:**
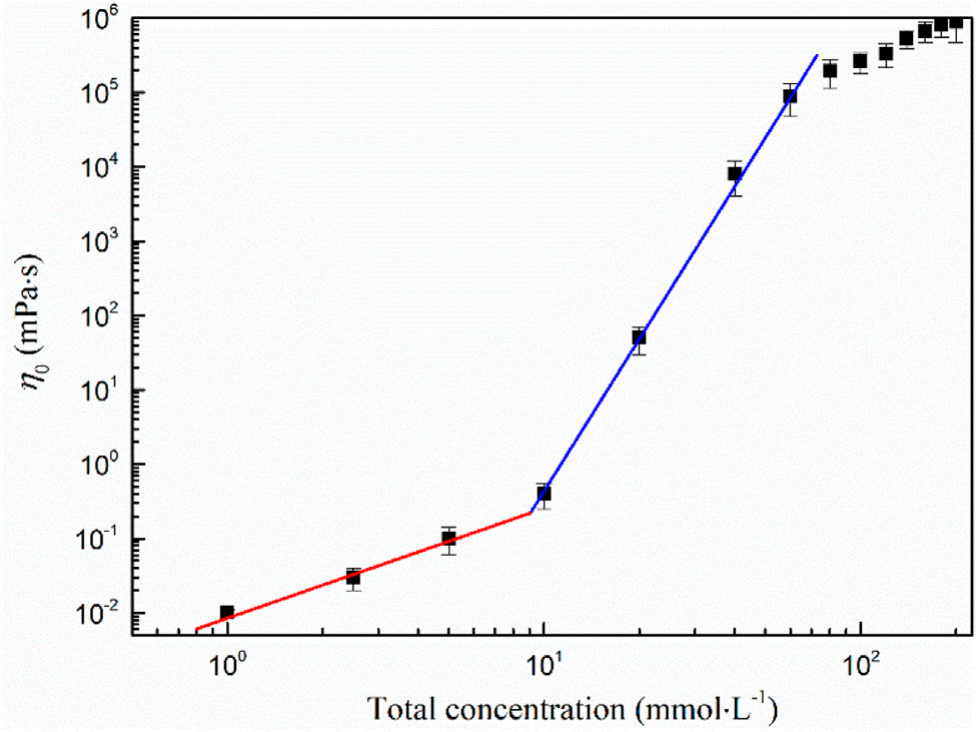
Shear viscosity versus total concentration of the MSA/CTAB system.

The critical overlapping concentration *C* ^*^of the wormlike micelle is 9.16 mmol·L^−1^, which can be obtained from the curve of the zero shear viscosity *η*_0_ versus the total concentration. This concentration is the critical surfactant concentration required for the wormlike micelles to entangle themselves (Liu et al., 2021b). As can be seen from Figure 4, the curve is divided into three regions by two points. When the concentration is less than *C* ^*^, *η*_0_ changes little with the concentration, showing a linear relationship, and follows Einstein equation $\eta_{0}=\eta_{water}\left(1+KC\right)$ (Dai et al., 2018). In this case, the surfactant in the solution is mainly in the form of small aggregates such as spherical micelles or short stick micelles, with a viscosity close to that of pure water. When the concentration is higher than *C* ^*^, *η*_0_ changes obviously with the concentration, and it increases exponentially and follows the law of proportion *η*_0_∼*C* ^P^, where the exponent *P* of the power function is constant. For the MSA/CTAB system, *p* = 2.76 is very close to *p* = 2.50 of the entangled wormlike micelles, which indicates that the surfactant micelles begin to form entangled aggregative wormlike micelles in the solution (Lu et al., 2022). When the concentration is more than 60 mmol·L^−1^, the power function law is still applicable, but *P* is reduced to 1.95. The *η*_0_ increases slowly with the concentration, which indicates that the branching point of the wormlike micelles of the system may occur and the branching behavior is strengthened. The appearance of the branching wormlike micelles is mainly due to the strong electrostatic shielding effect of CTAB in the system (Liu et al., 2021a).

**FIGURE 5:**
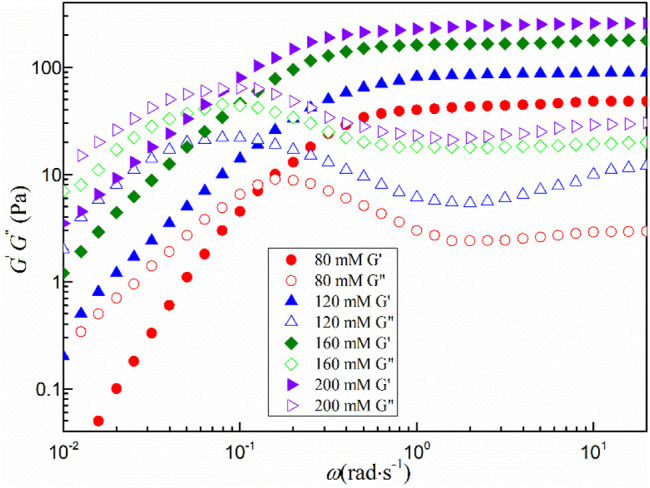
Dynamic shear rheological behavior of the mixed system.

Figure 5 shows the dynamic shear rheological behavior of the mixed system with a total concentration greater than 80 mmol·L^−1^. All viscoelastic fluids exhibit similar characteristics in the low angular frequency region. The storage modulus *G*′ is less than the loss modulus *G*″, the fluid mainly shows the viscous characteristics. When the angular frequency (*ω*) is larger than a certain critical value (*ω*_c_) and enters the high angular frequency region, the storage modulus *G*′ begins to be larger than the loss modulus *G*". The change of *G*′ is smooth and tends to a certain value, which is defined as the platform modulus *G*_0_. The transition from viscosity to elasticity indicates the formation of wormlike micelles in the fluid, which is consistent with the steady-state rheological results (Yan et al., 2020b).

**FIGURE 6:**
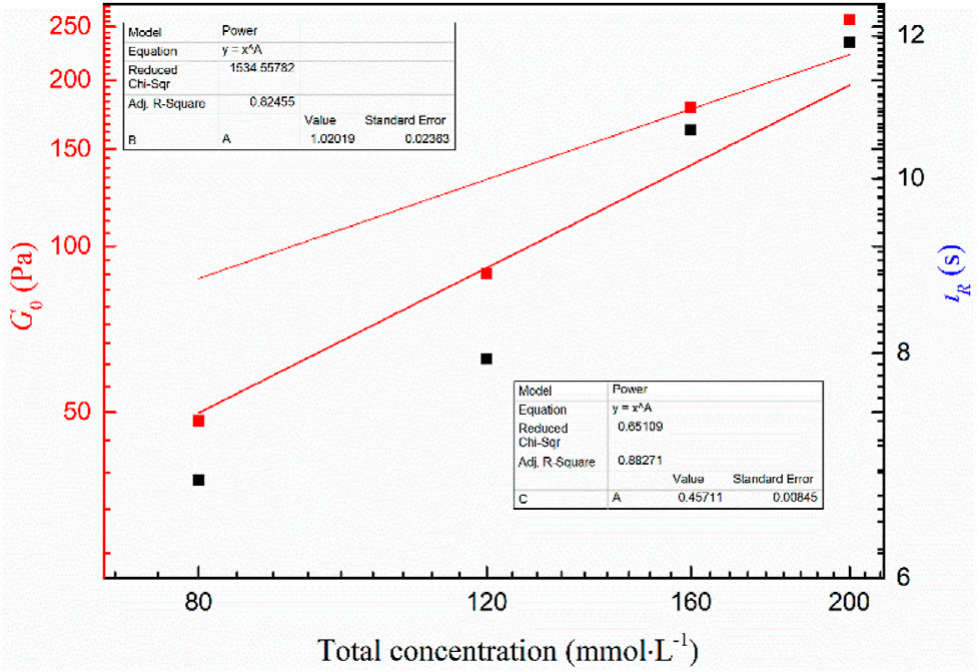
*G*_0_ and *τ*_R_ versus total concentration of the MSA/CTAB system.

In general, the viscoelasticity of wormlike micelles can be attributed to the fact that the entangled network of linear aggregates is amid a constant dynamic equilibrium of rupture and reorganization. It can be characterized by the relaxation time *τ*_R_ of the dynamic equilibrium, where the value is the reciprocal of *ω*_c_. As can be seen from Table 1, the *G*_0_ and *τ*_R_ of all viscoelastic fluids increase with increasing concentration. It can be seen from Figure 6 that the mixed system obeys the scaling law *G*_0_∼*C* ^m^ and *τ*_R_ ∼ *C* ^n^, where *m* and *n* are 1.02 and 0.45, respectively, which are close to the theoretical prediction values of branching wormlike micelles 1.8 and 0.25, respectively (Calabrese and Wagner, 2018). It is further demonstrated that the wormlike micelles developed branching behavior when the total concentration of the system increased at a later stage.

**TABLE 1:** Dynamic rheological parameters of the MSA/CTAB system at different concentrations.

*C*(mmol·L^−1^)	*G*_0_(Pa)	*ω*(rad·s^−1^)	*τ*_R_(s)
80	48.27	0.147	6.80
120	89.23	0.126	7.94
160	178.21	0.094	10.64
200	257.61	0.084	11.90

The above results show that the wormlike micelles formed by the non-covalent binding of MSA and CTAB molecules can resist the electrostatic effect of inorganic salts in the seawater. The wormlike micellar surface still has enough repulsive force to stabilize the three dimensional of the wormlike micellar network, resulting in excellent viscoelastic rheological properties in the seawater.

### Rheological Properties of pH-Responsive Wormlike Micelles

**FIGURE 7:**
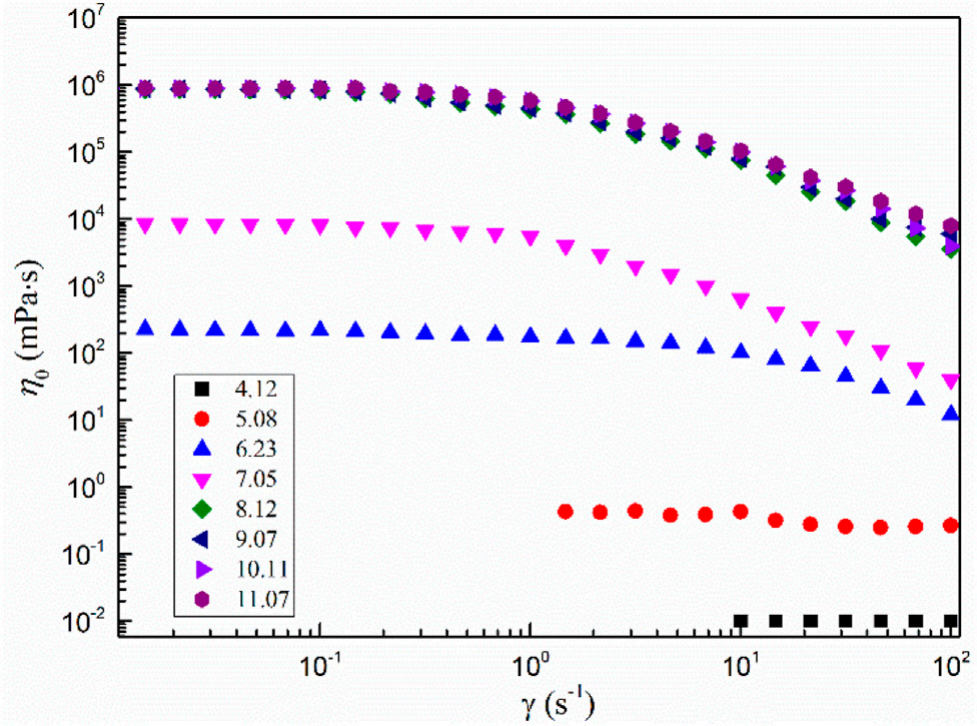
Steady-state rheological results of the 200 mmol L^−1^ MSA/CTAB mixed solution under different pH conditions.

Figure 7 shows the steady-state rheological test results of the 200 mmol·L^−1^ MSA/CTAB mixed solution under different pH conditions. When the pH value of the mixed solution is higher than 9.07, the *η*_0_ of the system does not change. The system still shows the rheological characteristics of shear thinning, and the solution is clear and transparent. When the pH value of the mixed solution decreased to 7.05, the system *η*_0_ decreased by four orders of magnitude. The viscosity of the mixed system decreased significantly, showing the typical Newton fluid behavior and losing the rheological characteristics of the wormlike micelle. The solution presented an opaque emulsion. When the pH value of the mixed solution continued to decrease to 4.12, fibrous crystals began to appear in the solution.

**FIGURE 8:**
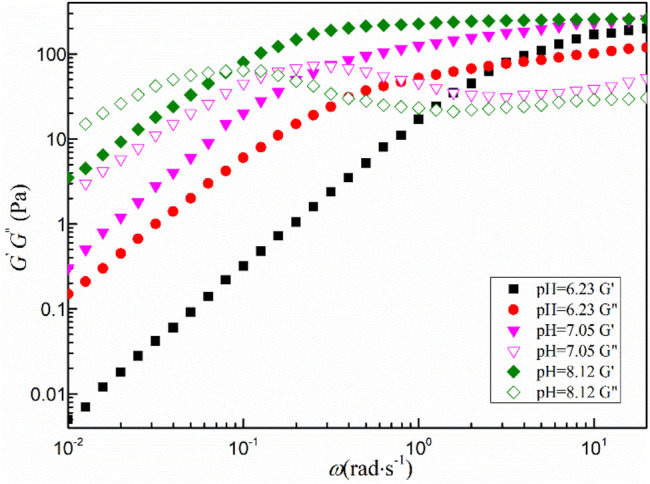
Variation of viscoelastic modulus of the MSA/CTAB mixed solution with oscillation angle frequency under different pH conditions.

Figure 8 shows the variation of viscoelastic modulus of the MSA/CTAB mixed solution with oscillation angle frequency (*ω*) under different pH conditions, which accords with the dynamic rheological characteristics of the wormlike micelles. It should be noted that, as the pH value of the mixed solution decreases, the solution undergoes a transition from elastic to viscous dominance. The intersection point of the viscosity modulus and the elastic modulus of the solution gradually moves toward the high angular frequency, which demonstrates the decrease of the relaxation time *τ*_R_. In general, the longer the relaxation time, the longer the wormlike micelles are, and vice versa (Walker, 2001).

**FIGURE 9:**
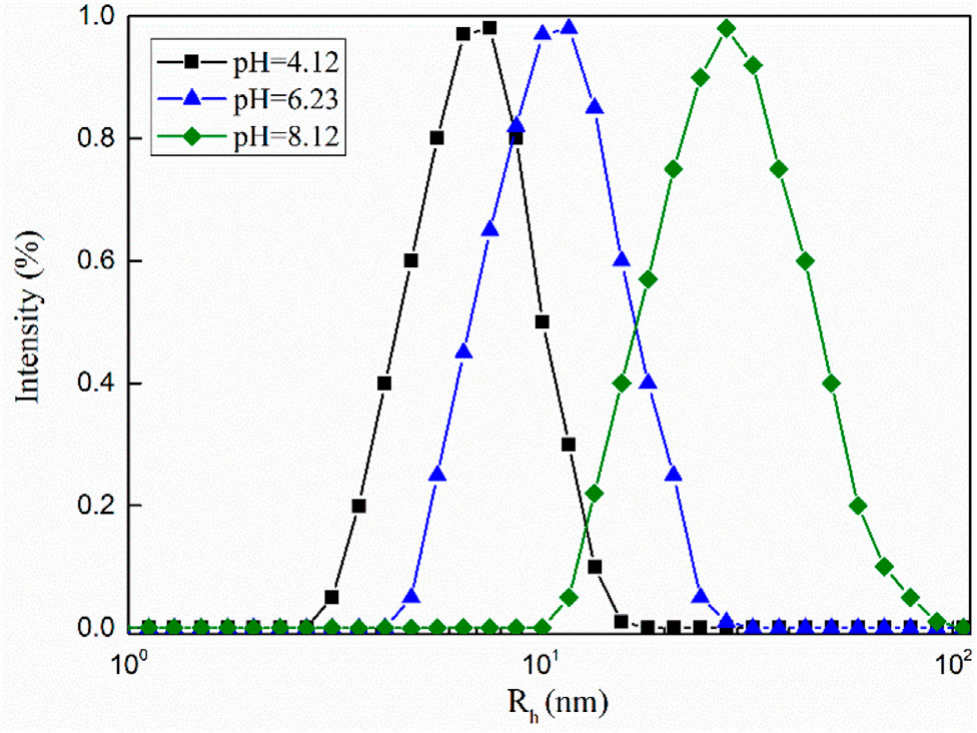
Hydrodynamic radius of the MSA/CTAB mixed solution under different pH conditions.

The measured hydrodynamic radius (*R*_h_) of the dynamic light scattering can reflect changes in the aggregate structure in solution (Jora et al., 2019). As shown in Figure 9, when the pH value of the mixed solution is equal to 8.12, the value of *R*_h_ is 28.2 nm, which shows that some longer wormlike micelles are formed in the system. As the pH value of the mixed solution decreased, the value of *R*_h_ also decreased. The dynamic light scattering test results show that the change in the macroscopic viscoelasticity of the mixed system is caused by the change in the structure of the micellar aggregates in the solution (Wu et al., 2018).

**FIGURE 10:**
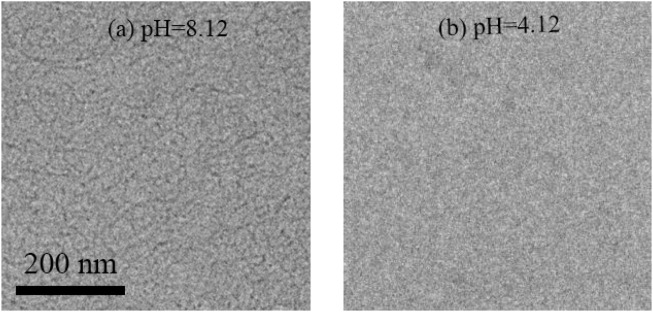
The micromorphology of the MSA/CTAB mixed solution at pH = 8.12 **(A)** and pH = 4.12 **(B)**.

To directly prove the change of aggregate structure in the mixed solution, the Cryo-TEM test was carried out to observe the micromorphology of the MSA/CTAB mixed solution with different pH values. As shown in Figure 10A, the Cryo-TEM image shows that the wormlike micelles do indeed form a network structure when the pH value of the mixed solution is equal to 8.12. The wormlike micelles disappear when the pH value drops to 4.12 in the Cryo-TEM image (Figure 10B). It is proved that the change from wormlike micelles to spherical micelles leads to the decrease of the apparent viscosity and the transition from Maxwell fluid to Newton-type fluid.

### Performance Evaluation of Fracturing Fluid With the MSA/CTAB System

**FIGURE 11:**
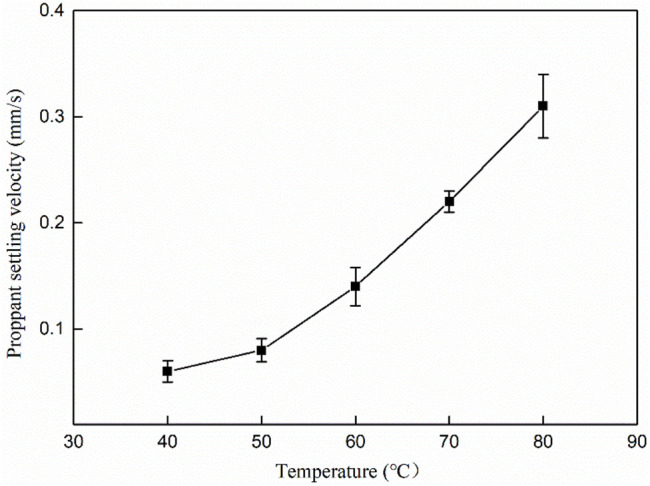
Settling curve of proppant in the MSA/CTAB mixed system.

The sand carrying capacity of fracturing fluid is an important index to evaluate whether proppant can be dispersed in fracturing fluid and keep non-subsidence for a certain time (Yegin et al., 2016). At present, the main evaluation method is to put proppant in fracturing fluid for a while to observe the distribution of proppant in the solution and the settling rate. Generally, the proppant settling velocity of less than 0.4 mm/s performs better. The proppant was selected as 20–40 mesh quartz sand (medium density, 1.65 g/cm^3^). The proppant was added into the mixed solution with a 30% mass fraction to test the proppant decreasing speed under different temperature conditions. Figure 11 is the settling curve of the proppant in the MSA/CTAB mixed system. It can be seen that the settling velocity of proppant sand in the system increases with the increase of temperature. When the temperature is 80°C, the settlement velocity is 0.31 mm/s, which can meet the requirement of oilfield construction. As the temperature increases, the molecule thermal motion dominates and the tangled network induced by the wormlike micelles is gradually disentangled; thus, the wormlike micelles are shortened in length, causing a decrease in viscosity of fracturing fluid.

**FIGURE 12:**
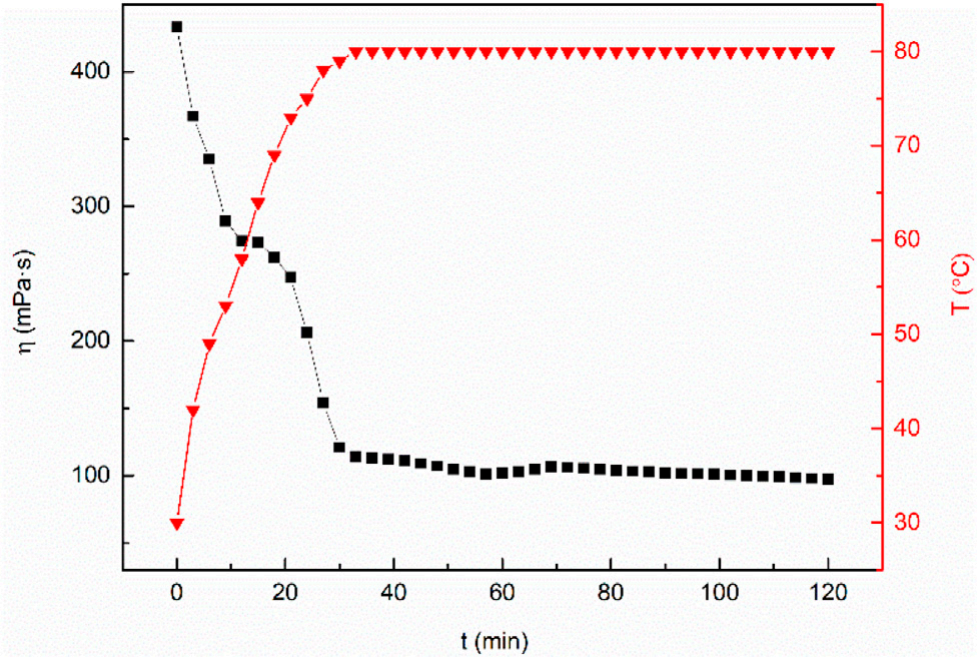
Change of viscosity of the MSA/CTAB system versus time showing the heat and shear resistance of the system.

The fracturing fluid is affected by mechanical shear and high temperature during construction, which leads to the micellar separation of wormlike micelles in clean fracturing fluid, and the performance of the fracturing fluid is decreased. Therefore, the temperature resistance and shear ability of fracturing fluid is an important index for evaluation (Zhao et al., 2017). The test temperature is set at 80°C and the shear rate is set at 170 s^−1^. As can be seen from Figure 12, the viscosity of the mixed system decreases gradually with the increase of temperature. The viscosity of the mixed system is almost constant with the increase of shear time when it reaches the test temperature. After 2 h of the constant shear test, the viscosity of the sample is still above 80 mPa·s, which meets China’s “General Specification for fracturing fluids-SY/T6376—2008”. Zhou developed a type of nanoparticle-modified clean fracturing fluid named VES-W. The viscosity of VES-W decreased to 34.9 mPa·s at 80°C (Zhou et al., 2022). Xiong synthesized a Gemini cationic C25-tailed surfactant named FL-25. The apparent viscosity of 3.06 wt% FL-25 solution at 80°C after 2 h shearing can remain approximately 70 mPa·s (Xiong et al., 2018). The above research results support the excellent temperature and shear resistance of the MSA/CTAB mixed system.

The gel-breaking property of fracturing fluid is the most crucial factor affecting the production of oil and gas well after fracturing operation. The fracturing fluid system with low residue content and low viscosity after gel breaking should be preferred (Yan et al., 2016). In this article, the pH response of the MSA/CTAB mixed system was used to break the gel, and citric acid was used as the gel breaker. A capsule breaker was prepared by coating a thin layer of shielding material on citric acid particles. The capsule breaker can ensure the viscosity requirement of fracturing fluid in the process of construction. After construction, the gel breaker can be released by the squeezing action of the formation fracture closing to break the gel quickly. As can be seen from Table 2, the viscosity of the MSA/CTAB mixed system under different temperatures is less than 5 mPa·s by using the capsule breaker with 0.1 wt%, which proves that the capsule breaker can break the gel effectively.

**TABLE 2:** Viscosity of the MSA/CTAB mixed system under different temperatures.

50°C	60°C	70°C	80°C
3.73 mPa s	2.98 mPa s	2.65 mPa s	2.11 mPa s

**FIGURE 13:**
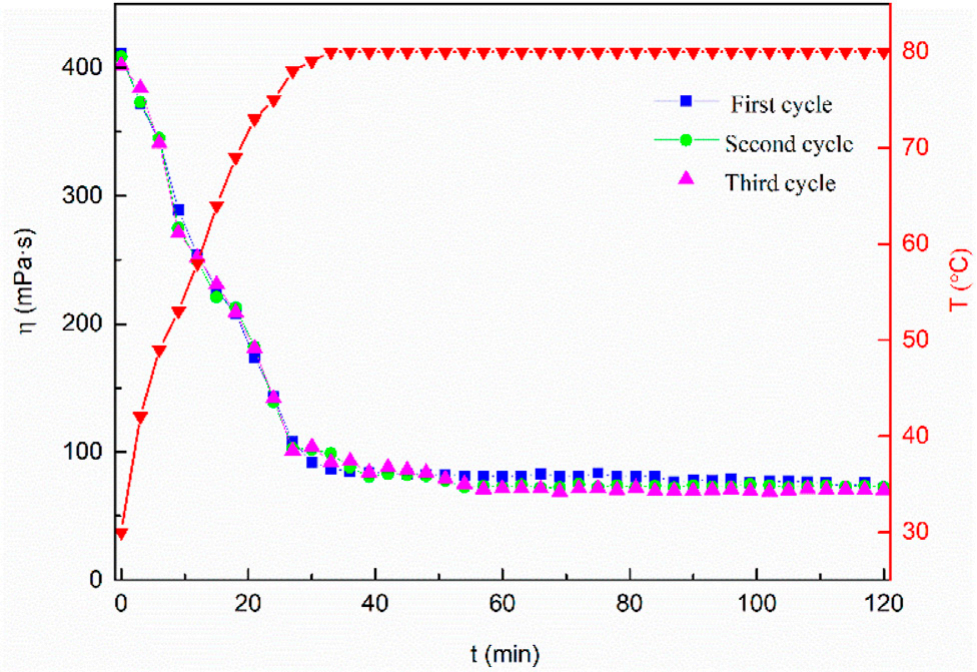
Change of viscosity of the MSA/CTAB cycled system versus time showing the heat and shear resistance of the system.

The mixed system concentration retention rate was measured using laboratory equipment to evaluate the fracturing fluid recycling performance. The related experimental results are shown in Table 3. As can be seen from Table 3, both MSA and CTAB have less loss and a higher retention rate in the simulated cores after simulated fracturing and backflow. This shows that if the MSA/CTAB mixed system was used for offshore fracturing, the cost of fracturing would be lower. At the same time, the temperature resistance and shear resistance of the mixed system circulation system at 80°C was investigated. The results are shown in Figure 13. The experimental results show that the viscosity of the circulation system after three cycles of gel forming-gel breaking is still higher than 30 mPa·s after 2 h of shearing, which can meet the requirement of carrying sand of clean fracturing fluid. It is proved that the system can meet the performance requirements of fracturing fluid for offshore re-fracturing.

**TABLE 3:** Related parameters of recycled cleaning fracturing fluids.

Number of Cycles	MSA Retention Rate (%)	CTAB Retention Rate (%)
1	92.75	86.85
2	85.47	79.26
3	81.51	72.51

## Conclusion

This article confirmed that the wormlike micelles formed by the non-covalent binding of MSA and CTAB molecules could resist the electrostatic effect of inorganic salts in the seawater. The excellent pH rheological responsiveness of the MSA/CTAB mixed system is due to the change of the self-assembled structure from wormlike micelles to spherical micelles. The results of the recycling performance evaluation show that the MSA/CTAB mixed system still meets the fracturing fluid performance requirements after three simulated recycling. It is hoped that the findings of this article will promote the application of clean fracturing fluid in oil fields.

## Data Availability

The original contributions presented in the study are included in the article/Supplementary Material, further inquiries can be directed to the corresponding author.
